# Age-related cortical thickness trajectories in first episode psychosis patients presenting with early persistent negative symptoms

**DOI:** 10.1038/npjschz.2016.29

**Published:** 2016-08-24

**Authors:** Carolina Makowski, Michael Bodnar, Ashok K Malla, Ridha Joober, Martin Lepage

**Affiliations:** 1 Integrated Program in Neuroscience, McGill University, Montreal, QC, Canada; 2 Douglas Mental Health University Institute, Department of Psychiatry, McGill University, Montreal, QC, Canada; 3 McGill Centre for Integrative Neuroscience, Montreal Neurological Institute, Montreal, QC, Canada; 4 Prevention and Early Intervention Program for Psychosis, Douglas Mental Health University Institute, McGill University, Montreal, QC, Canada

## Abstract

Recent work has clearly established that early persistent negative symptoms (ePNS) can be observed following a first episode of psychosis (FEP), and can negatively affect functional outcome. There is also evidence for cortical changes associated with ePNS. Given that a FEP often occurs during a period of ongoing complex brain development and maturation, neuroanatomical changes may have a specific age-related component. The current study examines cortical thickness (CT) and trajectories with age using longitudinal structural imaging. Structural T1 volumes were acquired at three time points for ePNS (*N*=21), PNS due to secondary factors (*N*=31), non-PNS (*N*=45) patients, and controls (*N*=48). Images were processed using the CIVET pipeline. Linear mixed models were applied to test for the main effects of (a) group, (b) time, and interactions between (c) time and group membership, and (d) age and group membership. Compared with the non-PNS and secondary PNS patient groups, the ePNS group showed cortical thinning over time in temporal regions and a thickening with age primarily in prefrontal areas. Early PNS patients also had significantly different linear and quadratic age relationships with CT compared with other groups within cingulate, prefrontal, and temporal cortices. The current study demonstrates that FEP patients with ePNS show significantly different CT trajectories with age. Increased CT may be indicative of disruptions in cortical maturation processes within higher-order brain regions. Individuals with ePNS underline a unique subgroup of FEP patients that are differentiated at the clinical level and who exhibit distinct neurobiological patterns compared with their non-PNS peers.

## Introduction

The first episode of psychosis (FEP) marks a critical turning point in the lives of affected youth and is manifested by varying combinations of symptoms at different levels of severity. Early persistent negative symptoms (ePNS) following a FEP are of particular interest due to their high correlation with poor functional outcome,^1,2^ including low clinical insight and deteriorating premorbid adjustment.^3^ However, ePNS has been seldom studied due to a wider focus granted to the emergence of later negative symptoms, especially in the course of schizophrenia. Such symptoms can be broadly categorized into general negative symptomatology, and primary, enduring negative symptoms (i.e. the deficit syndrome).^4,5^ Persistent negative symptoms, on the other hand, arguably cover a broader scope compared with the deficit syndrome,^4^ and allow for more flexibility in differentiating between primary and secondary negative symptoms over a shorter treatment period, as well as inclusion of less severe thresholds for negative symptom criteria.^6^ It should be noted that many studies do not make this distinction between primary or secondary, or refer to these terms without acknowledging limitations of the lack of etiological basis for such symptoms in the majority of clinical practice. Thus, the principal focus of this study will rest on the extraction of early PNS in a well-characterized clinical sample of FEP patients, including both affective and non-affective diagnoses.

From a neuroanatomical perspective, there is evidence to suggest there are gray matter abnormalities specific to patients with PNS.^7,8^ Although relatively fewer studies have been conducted to disentangle the neurobiological underpinnings of ePNS specifically, empirical evidence for progressive brain changes in FEP has been reported in several longitudinal studies^9,10^ and reviews.^11,12^ However, many studies have reported inconsistent, even null findings.^13–15^ Nesväg *et al.*^14^ commented that over a 5-year period, there were no meaningful differences in cortical thickness (CT), volume, or subcortical structures in chronic schizophrenia patients compared with healthy controls. A more recent study^15^ restricted their longitudinal investigation to a 3-year period, and commented that regardless of antipsychotic medication treatment, no CT changes could be observed in their sample of schizophrenia spectrum patients. It is clear there is still a great need to further disentangle the neural correlates of specific symptom constructs that are linked to poor prognosis and outcome, such as ePNS. Supporting this, a recent review^8^ reported disparities in reports of neurobiological changes underlying negative symptoms across different research groups. The review also encouraged future studies to distinguish between different types of negative symptoms after a FEP; for instance, comparing primary and secondary PNS (the latter having concurrent depressive, positive, and/or extrapyramidal symptomatology).^4,5^

Recent efforts have been put forth to delineate cross-sectional CT correlates of patient subgroups based on symptoms in schizophrenia and early psychosis. Nenadic *et al.*^16^ separated a sample of 87 schizophrenia patients into 3 groups (i.e. predominant negative, disorganized, and paranoid), reporting the most extensive cortical thinning in the negative symptom group. Morch-Johnsen *et al.*^17^ set their focus on persistent apathy, reporting thinner cortex within the left orbitofrontal and anterior cingulate cortices in apathetic FEP patients compared with the rest of their patient sample. Our group also reported CT findings within similar regions, in addition to elucidating patterns of cortical thinning within parahippocampal and superior temporal gyri, and the temporoparietal junction in FEP patients presenting with ePNS, compared with their non-PNS peers.^7^ However, all three of these studies were cross-sectional, thus no conclusions can be drawn about progressive changes in CT with respect to negative symptomatology.

It is important to note that many CT studies control for age within analysis, due to the well-documented normative progression of cortical thinning across age throughout adulthood.^18^ However, many studies reporting on FEP incorporate samples with ages spanning from adolescence to adulthood, without inquiring how reported findings change at this critical developmental transition. One exception can be found in the work of van Haren,^19^ probing the degree of cortical thinning attributable to normative aging processes in enduring schizophrenia, based on age of onset. A recent investigation by Pina-Camacho *et al.*^20^ also incorporated age effects within CT analyses applied to 196 FEP patients, and revealed cortical properties specific to an onset of psychosis before 20 years of age, where significant age effects were manifested differentially in a regional and diagnostic-specific manner. Hence, age is an important factor to consider when parsing apart cortical findings within different groups of FEP patients given ongoing neurodevelopment occurring during late adolescence/early adulthood.

This study aims to address differential progression of ePNS after a FEP at the neuroanatomical level, applying whole-brain CT analyses to a large sample of longitudinally followed FEP patients. First, the main effects of group (ePNS, non-ePNS, and Controls) and scan time (Baseline, 1- and 2-year follow-up (FUP1 and FUP2)) were examined using linear mixed models. The effect of age on CT in ePNS and non-ePNS patients was then examined, and compared with normative cortical thinning trajectories within a healthy control group. We also assessed specificity of CT results to the ePNS group by comparing to a subset of non-ePNS patients that presented with persistent negative symptoms due to secondary factors (sPNS). We hypothesized: (1) greater cortical thinning over time in the ePNS group compared with non-ePNS patients (including sPNS) and controls, in higher cognitive regions such as the prefrontal cortex, temporoparietal junction, and parahippocampal gyrus; (2) younger FEP patients with ePNS would show steeper rates of change in CT compared with non-ePNS patients; and (3) non-ePNS patients would show cortical thinning across age similar to healthy controls.

## Results

### Socio-demographic and clinical results

**Figure 1:**
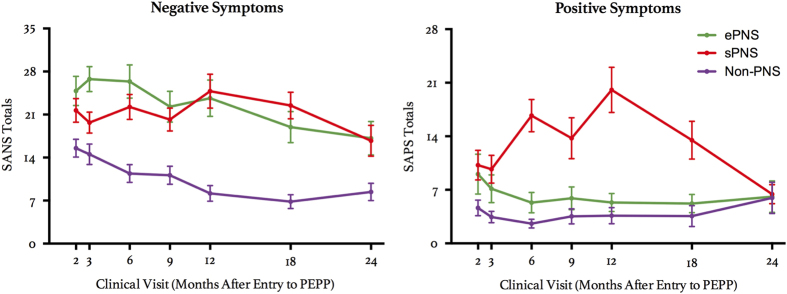
Mean Curves for SAPS/SANS scores across clinical visits in FEP subgroups. Data presented as mean±s.d. See Table 2 for corresponding statistics. ePNS, early persistent negative symptoms; FEP, first episode of psychosis; SAPS/SANS, scale for assessment of positive/negative symptoms; sPNS, persistent negative symptoms due to secondary factors.

In the FEP group, baseline scans were performed on average 4.0 (s.d.=1.9) months from entry into PEPP. For the entire group, including controls, inter-scan intervals were ~13.1 (s.d.=1.3) months between baseline and FUP1, and 12.6 (s.d.=1.7) months between FUP1 and FUP2. Nine participants (six FEP, three controls) were not scanned at FUP1, and had an average inter-scan interval of 27.0 (s.d.=3.2) months between FUP2 and baseline. The groups did not significantly differ in sex ratio, handedness, parental socioeconomic status (SES), or age at scanning time, as seen in Table 1. However, controls significantly differed from all patient groups on full-scale intelligent quotient (IQ) (not explained by test version) and years of education. Within the three patient groups, there were no significant differences in Calgary Depression Scale for Schizophrenia (CDSS) scores, time elapsed between the magnetic resonance imaging (MRI) scan and symptom evaluation across all scanning time points, nor duration of untreated psychosis (DUP)/illness. Amount of antipsychotic prescribed and anxiety levels (as assessed by the Hamilton Anxiety Rating Scale (HARS)) were significantly higher for sPNS patients, compared with non-PNS, only at Scan 2. With respect to negative symptoms, the ePNS and sPNS groups had higher scores on the Scale for the Assessment of Negative Symptoms (SANS) compared with other non-PNS patients across all time points. The sPNS patient subgroup also had significantly higher scores on the Scale for the Assessment of Positive Symptoms (SAPS) compared with the other two patient groups across most clinical time points (with the exception of the last 2-year FUP assessment). See Figure 1 for depiction of SAPS/SANS score for each FEP subgroup across clinical time points and Table 2 for corresponding generalized estimating equation statistics. Finally, no significant association between DUP and SANS totals pertaining to each scan time point was observed (Scan 1: *r*=−0.095, *P*=0.41; Scan 2: *r*=0.041, *P*=0.71; Scan 3: *r*=0.14, *P*=0.27).

### CT analyses

#### Main effect of group

**Figure 2:**
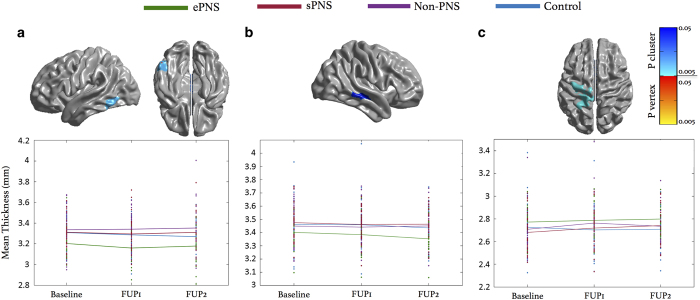
Main effects of group and time. (**a**) Significant cortical thinning in ePNS (main effect of group) compared with the whole non-PNS group was observed in left inferior temporal region (136 df). (**b**) ePNS group shows significant cortical thinning from baseline to FUP2 in right middle temporal gyrus (52 df). (**c**) Increases in cortical thickness from Baseline to FUP1 are seen in the non-PNS group within the left dorsal precentral and post-central gyri (137 df), with this increase largely driven by the sPNS subgroup. Blue color bar represents significant RFT-thresholded clusters (no significant results at the vertex level). All statistics are projected on an average surface template generated from the analyzed sample. Plot of mean thickness of peak cluster depicted per group across three scanning time points. ePNS, early persistent negative symptoms; FUP, follow-up; RFT, random field theory; sPNS, persistent negative symptoms due to secondary factors.

There was a significant cluster of cortical thinning in ePNS patients compared with the entire non-ePNS sample, within the left inferior temporal/fusiform gyrus, (Brodmann Area (BA) 37). No significant group differences between controls and FEP groups were found. See Figure 2a for details and Supplementary Figure 1 for *t*-statistic maps.

#### Main effect of time point

The ePNS group exhibited significant cortical thinning from Baseline to FUP2 within right middle temporal gyrus (BA 22). The non-ePNS group displayed increased CT from Baseline to FUP1 in left dorsal pre- and post-central gyri (BA 1–5), which on further analysis was found to be driven by the sPNS subgroup. See Figure 2b,c for details and Supplementary Figure 2 for *t*-statistic maps.

#### Group by time interaction

No significant group×time interaction on CT was found for any contrasts comparing ePNS and non-ePNS patients and controls.

#### Group by age interaction: linear effects of age

**Figure 3:**
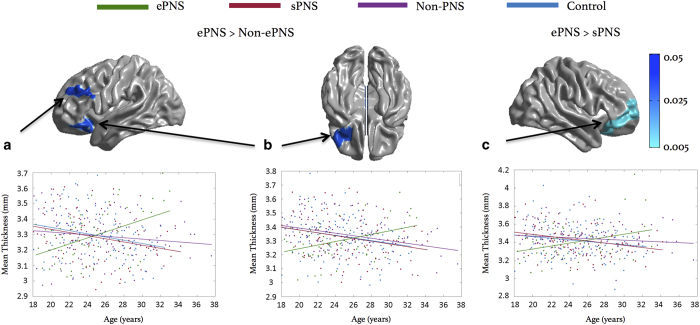
Age×group interaction: linear effects of age. Significant prefrontal ROIs extracted from examination of significant age×group interaction effect, in relation to ePNS group. ROIs (**a** and **b**) compare ePNS and entire non-PNS group, whereas (**c**) compares ePNS and sPNS subgroups. Plots of mean cortical thickness for each ROI comparing all three patient groups and controls depicted directly below each RFT-thresholded brain map. Omnibus statistics for ROIs are as follows: (**a**) F_(3,371)_=9.09, *P*<0.001; (**b**) F_(3,371)_=6.49, *P*<0.001; (**c**) F_(3,371)_=5.26, *P*<0.001. *Post hoc* analyses revealed that the ePNS group had a significantly different regression slope from all other groups (controls, sPNS, and non-PNS without sPNS), with all *P*’s ⩽0.001. Annual rates of change after age 18 in Controls, sPNS, and Non-PNS groups amount to ~0.32% CT loss per year across the prefrontal ROIs. By contrast, ePNS patients showed an annual increase in CT of 0.37% per year. Blue color bar represents significant RFT-thresholded clusters with *P*-corrected=0.05 (no significant results at the vertex level). All statistics are projected on an average surface template generated from the analyzed sample. ePNS, early persistent negative symptoms; RFT, random field theory; sPNS, persistent negative symptoms due to secondary factors; ROI, region-of-interest.

Widespread frontal regions showed an interaction effect of age×group, such that the ePNS group showed a positive effect with age on CT compared with the non-ePNS sample. Further exploration using a reduced threshold of *P*-uncorrected=0.01 revealed significant clusters of increased CT with age within left dorsolateral prefrontal cortex (DLPFC, BA Area 9) and left inferior orbitofrontal cortex (BA 10/11) in the ePNS group compared with non-PNS. When directly comparing the ePNS and sPNS patient groups, ePNS showed a significantly different and positive relationship with age in right anterior frontal regions/orbitofrontal gyrus (BA 10/11) with *P*-uncorrected=0.005. Mean CT was extracted across vertices comprising (1) left DLPFC, (2) left- and (3) right anterior/orbital frontal clusters. Regression slopes depicting the relationship between age and mean CT for all three ROIs revealed a significantly different and positive slope for ePNS compared with sPNS, non-PNS patient groups, and controls. Figure 3 illustrates clusters thresholded with random field theory (RFT) and corresponding plots. Annual rates of change detailed in Supplementary Table 1. No other group contrasts showed a significant linear interaction with age, with the exception of direct comparisons between sPNS and the remaining non-PNS patients, where the sPNS group showed significant CT increases with age in the left post-central gyrus compared with remaining non-PNS patients, as depicted in Supplementary Figure 3.

#### Group by age interaction: quadratic effects of age

**Figure 4:**
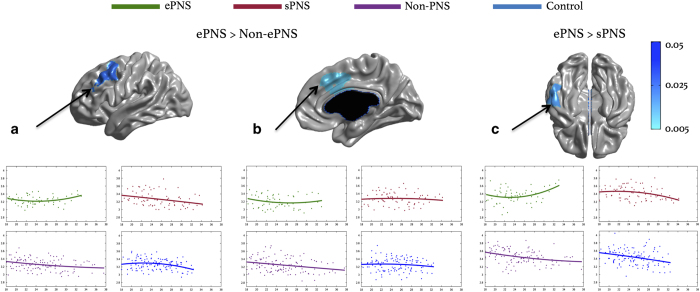
Age^2^×group interaction: quadratic effects of age. Significant prefrontal ROIs extracted from examination of significant age^2^×group interaction effect, in relation to ePNS group. ROIs (**a** and **b**) compare ePNS and entire non-ePNS group, whereas (**c**) compares ePNS and sPNS subgroups. Each of the three ROIs represent regions of cortical thickness with significantly different and positive quadratic relationships with age in the ePNS group only, with *P*-corrected<0.05. Blue color bar represents significant RFT-thresholded clusters with *P*-corrected=0.05 (no significant results at the vertex level). All statistics are projected on an average surface template generated from the analyzed sample. ePNS, early persistent negative symptoms; RFT, random field theory; sPNS, persistent negative symptoms due to secondary factors; ROI, region-of-interest.

Significant group×age^2^ interactions on CT were found between the non-ePNS and ePNS patient groups, such that the ePNS group showed significantly positive quadratic effects of age within the right middle cingulate (BA24, extending dorsomedially) with *P*-uncorrected=0.005, and a more posterior cluster of the left DLPFC, encompassing pre-supplementary motor area (independent of the first DLPFC cluster described with predominantly linear effects of age) with *P*-uncorrected=0.01, in comparison to non-ePNS patients and controls. Furthermore, a significant cluster emerged within the left inferior temporal gyrus (BA 37) with a significantly different and positive quadratic relationship of CT with age in the ePNS group compared with the sPNS group. See Figure 4 for thresholded maps and plots, and Supplementary Figure 4 for *t*-statistic maps. These three regions were all better explained by inclusion of a quadratic age term, as opposed to linear, as indicated by comparison of Akaike Information Criterion (AIC) values and significance testing with likelihood ratio tests. See Supplementary Table 2. Furthermore, as seen in Supplementary Table 3, significant results were not altered after covarying for antipsychotic dosage.

## Discussion

The current study examined structural neuroanatomical patterns in clinically well-characterized FEP patients with ePNS between the ages of 18 and 35. Significant differences in CT were found at the group level, across a FUP2 period, and longitudinally as a function of age, compared with other non-ePNS patients and controls. Patients with ePNS showed significantly thinner cortex within the left inferior temporal gyrus compared with non-ePNS patients, and time-specific cortical thinning over a 2-year period in the right middle temporal cortex. Observed changes within bilateral temporal cortices corroborate previous studies implicating progressive brain changes within the temporal lobes in FEP,^9,10^ and further lend support to previous findings from our research group examining the ePNS construct and its unique neuroanatomical correlates.^7,21,22^ On the other hand, non-ePNS patients showed a different pattern of CT change over FUP1, with increased CT in left motor areas (i.e., dorsal pre- and post-central gyri), driven by the sPNS subgroup. This CT increase is speculated to occur as a consequence of positive symptoms, which has been supported previously by findings of a positive correlation between post-central gyri-volumes and SAPS scores.^23^

We also explored the effects of age on CT in FEP patient subgroups, motivated by the idea that neurodevelopmental processes are ongoing throughout late adolescence and early adulthood, and may be altered in patients. Linear and quadratic models of age yielded clear differential patterns of CT across age unique to ePNS, such that CT increased linearly with age in left DLPFC and bilateral orbitofrontal cortices in this group, and positive quadratic age effects were found within a second left DLPFC cluster (encompassing pre-supplementary motor area), right middle cingulate (extending dorsomedially), and left inferior temporal cortex. In a similar vein of exploration, Ecker *et al.*,^24^ reported significantly opposing CT trajectories with age in a sample of youth with autism spectrum disorder (ages 7–25), finding comparable linear and quadratic age×group interaction effects, with decreased thickness at younger ages, but significantly increased thickness in autistic adults.

The reported age-related CT changes in relation to ePNS in a sample of longitudinally followed FEP patients merit careful examination of potential biological markers and mechanisms underlining neurodevelopment in ePNS in future studies. The CT metric utilized in the current study underscores a biological inference of cellular gray matter composition comprising the thickness of the cortical mantle. Seminal studies of CT and gray matter volume trajectories in normative development emphasize the late-maturational time course of the frontal lobes;^25,26^ specifically, the DLPFC is under dynamic refinement for longer than was initially cited, developing well into the second decade of life.^27^ Cortical maturation processes are largely steered by synaptic pruning and reorganization of synaptic connections, as well as myelination of fibers near the gray–white matter boundary,^28^ which may be altered in schizophrenia.^29,30^ Furthermore, increased CT with age may share some overlap with the early hypothesis of delayed brain maturation driving the characteristic onset of schizophrenia in adolescence,^31^ albeit specific to ePNS based on our findings.

Insight into understanding the neurobiological mechanisms underlying CT changes may also be gained from the recent findings implicating neuroinflammation in schizophrenia.^12,32^ It has been proposed that at acute phases of illness, neuroinflammation may lead to global and local brain swelling.^12^ Although the neurobiological mechanisms underlying CT changes are difficult to elucidate using MRI, our findings demonstrate that FEP patients with ePNS represent a clinically and neuroanatomically distinct subgroup of patients, and future studies are strongly encouraged to consider age as a significant factor when examining progressive brain changes in FEP.

At the clinical level, further support for our delineation of three separate subgroups of FEP patients was found in the significantly different longitudinal symptom profiles, namely when examining negative and positive symptoms. Relationships between negative symptoms pertaining to each scanning time point and duration of untreated psychosis were further explored, given previous literature indicating DUP as a potential predictor of negative symptomatology.^33–35^ Our own data did not uncover any differences in DUP between our FEP subgroups, nor a relationship between DUP and negative symptoms, consistent with some other studies,^36,37^ and also speaks to the potential non-linear relationship between these two variables.^35^

The present study has several strengths and limitations. It is based on a large sample of FEP patients that were recruited from a well-defined catchment area without competing other services, thus it is likely to reflect a sample with minimal recruitment biases. Furthermore, the sample was followed longitudinally up to 2 years after the patient experienced a FEP, alongside reliable clinical information. However, a large proportion of patients were taking antipsychotic medication throughout the study. Although there were no differences between ePNS and other patients in antipsychotic dosage/adherence, several studies have alluded to cortical changes accompanying antipsychotic use.^19,38–40^ However, an additional analyses controlling for antipsychotic medication did not diminish our significant results. It should also be noted that the ePNS group was smaller than the other patient groups, and this may have contributed to the lack of difference between our FEP subgroups on sex, as has been reported previously in the PNS literature.^41^ However, it should be noted that the recruited sample already held a strong male bias, and thus it proved difficult to detect sex differences specific to PNS. For the neuroimaging analyses, to accommodate potential loss of power in the ePNS group, a slightly lower threshold was required to detect some of the significant effects reported with respect to the age×group interactions. We also did not find widespread cortical thinning comparing our ePNS and non-ePNS groups to controls, which is discordant with other reports of CT differences in FEP. In addition to heterogeneity within and across reported samples, the inconsistencies found within such investigations could be partially attributed to utilization of ROIs (which are largely dependent on the pipeline used), rather than whole-brain vertex-wise approaches, or the use of different covariates in statistical models, such as controlling for age. This latter point deserves recognition, as the time at which a FEP occurs is highly non-trivial in both social and neurodevelopmental contexts. Although best efforts were put forward to reduce noise in the imaging data and retain only high-quality scans, it is acknowledged that even subtle head motion can significantly alter CT findings, as supported by recent work,^42,43^ which may also contribute to the explanation as to why previous studies have reported exaggerated cortical thinning in FEP samples compared with controls. Finally, caution should be exercised when interpreting the elements underlying persistent negative symptoms due to secondary factors. Our current dataset does not allow us to parse apart the etiology of symptoms, and thus we cannot say with confidence whether the persistent negative symptoms we observe are indeed primary or secondary in relation to other symptom constructs. The current sample is also limited in its definition of extrapyramidal symptoms (EPS), as patients are only identified with EPS if they are on anticholinergic medication. Thus, the sPNS sample presented in this manuscript may be an underestimate.

Progressive brain changes following a FEP are non-uniform across patients, as demonstrated by the presented findings implicating ePNS and age as significant contributing factors to underlying neuroanatomical variations in the early course of psychosis. It has not escaped our attention that ePNS patients are akin to Tim Crow’s initial conception of ‘type II schizophrenia’ in 1980,^44^ warranting further investigation due to the clear poor functional outcome associated with these symptoms.^2^ The current study highlights the power of using a symptom-based classification approach as opposed to a diagnostic approach, to elucidate dynamic cortical changes across time. Such approaches may yield more fruitful results when linking biomarkers and their progression after a FEP to clinical outcome, which could further be used to inform clinical practice and psychotherapies.

## Materials and methods

### Participants

All patients were recruited from the Prevention and Early-Intervention Program for Psychoses (PEPP-Montreal), at the Douglas Institute in Montreal, Canada, and were part of a longitudinal naturalistic outcome study. PEPP is a specialized early-intervention service for individuals between the ages of 14 and 35 who have recently experienced a FEP within a local catchment area of Southwest Montreal. Details are outlined in ref. 45. Briefly, the program involves a comprehensive approach with intensive medical and psychosocial interventions provided within the context of a modified assertive case management program. Inclusion criteria at PEPP include a diagnosis of affective or non-affective psychosis, an IQ >70, and no past antipsychotic medication treatment for >1 month.

### Neuroimaging component

The neuroimaging study began in 2003 and has spanned over a decade, comprising three scheduled visits: baseline, FUP1, and FUP2. Forty-two patients and forty-six controls dropped out of the study after baseline, leaving a total of 100 patients and 48 healthy controls with at least 2 scans. Three patients were not included in subsequent analysis due to insufficient longitudinal symptom data (two cases) or non-compliance to the time line of the study (one case). Only individuals of age ⩾18 were considered for the neuroimaging portion of the study, as well as clinically stable status and no major medical disorders. Exclusion criteria included a history of neurological illnesses and head trauma, resulting in loss of consciousness that could affect cognition, presence of neurological disorder determined by medical record examination, lifetime diagnosis of substance dependence, and/or any potential contraindication for the MR scan.

Non-clinical healthy controls were recruited through advertisements within the same local catchment area. In addition to exclusion criteria listed for FEP patients, controls were excluded if they had any current/past history of Axis I disorders, and/or a first-degree family member suffering from schizophrenia or related schizophrenia spectrum psychosis. All participants provided written informed consent, and the research protocol was approved by the Douglas Mental Health University Institute human ethics review board.

### Clinical assessment and demographic data

Diagnosis for each patient was made on the basis of structured clinical interview for DSM-IV,^46^ performed by a trained interviewer, and confirmed by at least one senior psychiatrist (R.J. or A.K.M.). Depression and anxiety symptoms were assessed with the CDSS^47^ and HARS,^48^ respectively. Negative and positive symptoms were assessed with the SANS^49^ and SAPS.^50^ DUP was also assessed, referring to the period of time in weeks between onset of psychotic symptoms to adequate treatment with antipsychotics, as described elsewhere.^51^ Antipsychotic medication dosages were converted to chlorpromazine equivalents according to the literature,^52^ and multiplied by percent medication adherence.^53^ For both controls and patients, parental SES was estimated using the Hollingshead SES Rating Scale,^54^ and handedness determined with the Edinburgh Handedness Inventory.^55^ Due to a change in the neuropsychological test battery used mid-way through the study, full-scale IQ was assessed with the Weschler Adult Intelligence Scale (WAIS-III) for a proportion of subjects, and the Weschler Abbreviated Scale of Intelligence (WASI) for the remaining sample.^56,57^

Early PNS were defined according to the following criteria: (1) global rating of moderate or more on at least one negative symptom as measured by the SANS, (2) global rating of mild or less on all positive symptoms as measured by the SAPS, (3) a total score of ⩽4 on the CDSS, (4) absence of EPSs requiring anticholinergic treatment, and (5) all above criteria are continuously met for a period of at least 6 months.^5,7^ Patients were classified as having PNS due to secondary factors, or sPNS, if criteria 2, 3, and/or 4 were not met.

### MRI acquisition

All scanning was carried out at the Montreal Neurological Institute (MNI) on a 1.5 T Siemens Sonata whole body MRI system (Siemens Medical Systems, Erlangen, Germany). Structural T1 volumes were acquired for each participant using a three-dimensional gradient echo pulse sequence with sagittal volume excitation (repetition time=22 ms, echo time=9.2 ms, flip angle=30, 180 1 mm contiguous sagittal slices). The rectangular field-of-view for the images was 256 mm (SI) and 204 mm (AP). Information about quality control (QC) is detailed in Supplementary Information.

### Post-processing

All raw scans that passed QC were submitted to the CIVET pipeline (Version 2.0.0: http://www.bic.mni.mcgill.ca/ServicesSoftware/CIVET).^58,59^ Detailed steps have been described by our group elsewhere^7^ and include: (1) registration of T1-weighted images to a standardized space and correction for non-uniformity artefacts, (2) parcellation of gray matter, white matter, cerebral spinal fluid, and background noise, (3) extraction of high-resolution gray and white matter surfaces comprised of 40,962 vertices within each hemisphere, (4) non-linear registration of cortical surfaces to a high-resolution template for inter-subject correspondence of vertices, (5) reverse transformation (initially done in step 1) to estimate CT in native space for each subject using the t-link metric, and (6) smoothing the data with a 30-mm kernel, which has been previously shown to be an optimal level of smoothing when utilizing the t-link metric.^60^ All volumes, with the exception of one scan, passed through the pipeline and QC process, due to minimization of error within initial QC of raw scans. All CIVET outputs were quality controlled using the CBRAIN platform,^61^ of which significant mask errors and/or minor pipeline errors were corrected through in-house scripts if feasible. See Supplementary Information for quality control procedures.

### Statistical analyses

Demographic and clinical variables (with a single time point) were analysed with one-way analysis of variance for continuous variables or Kruskal–Wallis *H*-tests for nominal variables. For IQ, an analysis of covariance was used to covary for test version. For SAPS/SANS, generalized estimating equations were used to assess differences between FEP groups across clinical time points. Antipsychotic dosages, CDSS scores, and the time period in months between scan and nearest symptom evaluation were assessed between the three patient groups at each scan-time point, using one-way analysis of variances for normally distributed variables (*post hoc* Tukey’s honest significant difference test), and Kruskal–Wallis *H*-tests for nonparametric variables (*post hoc* Mann-Whitney *U*-tests). Relationship between SANS totals at each scanning time point and DUP was estimated using Spearman’s rank correlations. Analyses of clinical variables were conducted using PASW Statistics 21 (SPSS, 2009, Chicago, IL, USA) and were two-tailed with a critical *P*-value of 0.05.

### CT analyses

To assess differences in CT between ePNS, non-PNS, and healthy control groups, statistics were performed across all 81,924 vertices of the cortical surface using the SurfStat toolbox within Matlab (http://www.math.mcgill.ca/keith/surfstat/). First, the main effect of group was tested using the linear mixed effects model outlined below, controlling for age, sex, handedness, and a proxy measure of Brain Volume (ProxyBV):

$$Y=\mathrm{intercept}+d_{1}+\beta_{1}\left(\mathrm{Group}\right)+\beta_{2}\left(\mathrm{Age}\right)+\beta_{3}\left(\mathrm{Sex}\right)+\beta_{4}\left(\mathrm{Handedness}\right)+\beta_{5}\left(\mathrm{ProxyBV}\right)+\mathrm{random}\left(\mathrm{Subject}\right)+\varepsilon$$

where *Y* represents CT, *d*_1_ is the random within-subjects effect, *β*_1-5_ represent regression coefficients, and *ε* is residual error. ProxyBV (subcortical gray matter+white matter+cerebrospinal fluid volume) was entered as a covariate, based on rationale provided by ref. 29. Briefly, this variable excludes GM volumes, which would otherwise account for ~40% of total intracranial volume and is highly correlated with mean CT. The proxy measure of brain volume bypasses the potential of removing the effect of interest, while still controlling for important confounds associated with brain volume. To test for a main effect of time, data were filtered to examine each group separately, and ‘*β*_1_(Group)’ was replaced with ‘*β*_1_(Time)’ in the mixed effects model. To test for the interaction between scan time (Baseline, FUP1 and FUP2) and group, two additional terms were added: ‘*β*_6_(Time)’ and ‘*β*_7_(Group×Time)’.

Finally, we tested the interaction between age and group membership on CT. Due to the idea that CT may not change linearly over time within an individual^62,63^ linear and quadratic effects of age (i.e. age, age^2^) were included in the mixed effects models. Higher-order polynomial terms were not tested. The age variable was mean-centered for all analyses. We used a forward selection approach; that is, the simplest model was tested for first (linear age effect). For linear age effects, ‘*β*_6_(Group×Age)’ was added to the original model outlined above. For quadratic effects of age, additional terms were incorporated: ‘*β*_7_(Age^2^)’ and ‘*β*_8_(Age^2^×Group)’, and tested for separately. To test whether a linear or quadratic age term provided the best fit for significant regions, parameters of each model were estimated with theoretical likelihood ratio tests. To compare different models of age directly, the AIC was applied to obtain log-likelihood values.^64^ The model with the best fit for the region tested has a smaller AIC value. For all analyses, statistical maps were thresholded and multiple comparisons were taken into account using RFT for non-isotropic images, with an uncorrected *P*-value of *P*=0.005.^65^ This procedure is implemented within SurfStat and limits the chance of reporting a false positive finding to <0.05. Given the conservative nature of RFT, exploratory analyses were also performed using a more liberal uncorrected threshold, *P*=0.01. For group comparisons, analyses were initially run contrasting ePNS and all non-ePNS patients. A subsequent round of analyses then examined potential differences between sPNS, ePNS, and remaining non-PNS patients. Finally, to ensure any significant results could not otherwise be explained by exposure to antipsychotic medication, a covariate was added to all linear mixed effects models presented above to control for cumulative chlorpromazine equivalent dosages multiplied by medication adherence at each patient’s scan.

## Figures and Tables

**Table 1:** Demographic and clinical information for longitudinal sample

	*FEP*											
			*Non-ePNS*									
	*ePNS*		*sPNS*		*Non-PNS*		*Controls*					
	N	*%*	N	*%*	N	*%*	N	*%*	*Statistic*	*df*	P*-value*	Post hoc
*General demographics and clinical information*												
*N* (and % having all 3 scans)	21	86	31	52	45	58	48	58				
Male	15	71	22	71	32	71	28	58	*χ*^2^=2.4	3	0.5	—
Right handed	17	81	26	84	39	87	42	88	*χ*^2^=0.6	3	0.9	—
Diagnosis												
SCZ/SCZ-F	16	76.2	27	87.1	25	55.6						
Affective disorder	3	14.3	1	3.2	15	33.3						
Delusional disorder	0	0.0	1	3.2	2	4.4						
Psychosis NOS	2	9.5	2	6.5	3	6.7						

	*Mean*	*s.d.*	*Mean*	*s.d.*	*Mean*	*s.d.*	*Mean*	*s.d.*				
Education in years	11.1	2.5	11.7	2.4	12.6	2.4	14.2	2.5	F=10.8	3, 144	<0.0001	1, 2, 3 < 4
Socioeconomic status	3.4 [16]	1.0	3.3 [30]	1.2	3.0 [42]	1.0	3.3 [45]	0.9	*χ*^2^=5.4	3	0.1	—
Full scale IQ^a^	96.9	15.3	98.4	17.2	100.3	14.1	111.1 [46]	14.8	F=6.9	3, 142	<0.0001	1, 2, 3 < 4
DUP (weeks)	45.4 [20]	59.2	57.1	100.5	83.6 [43]	170.5			*χ*^2^=0.1	2	0.9	—
DUI (years)	5.3 [20]	4.4	7.8 [30]	6.5	7.2 [42]	7.1			*χ*^2^=1.2	2	0.6	—

*Scan 1*												
Age	23.2	3.6	24.7	4.1	24.6	4.6	23.8	3.4	F=0.9	3, 144	0.4	—
SANS totals	25.4	9.3	23.2	12.0	14.2	10.5			F=10.5	2, 96	<0.0001	1, 2>3
*Affective flattening*	7.5	6.1	7.7	6.0	4.4	4.8			F=4.0	2, 96	0.021	1, 2>3
*Alogia*	3.2	2.7	2.1	3.4	1.0	1.9			*χ*^2^=10.5	2	0.005	1>3
*Avolition/apathy*	6.8	2.3	5.8	3.2	3.8	2.7			*χ*^2^=18.0	2	<0.001	1, 2>3
*Anhedonia/asocality*	7.9	2.5	7.6	4.1	4.8	3.5			F=8.3	2, 96	<0.001	1, 2>3
SAPS totals	8.2	10.2	17.3	15.1	4.0	5.4			*χ*^2^=25.5	2	<0.0001	2>1, 3
CDSS	2.4	2.7	3	3.2	1.7 [44]	2.4			*χ*^2^=3.2	2	0.2	—
HARS	4.2	5.1	5.5	5.9	3.5	5.2			*χ*^2^=5.22	2	0.074	—
CPZ equivalent (in mg)	758.4	671.3	944.7	838.8	776.2	700			*χ*^2^=0.6	2	0.7	—
Adherence (%)	86.6	21.3	86.2	20.9	84.9	27.3			*χ*^2^=0.4	2	0.8	—
|Scan - Symptom Eval| (mo)	0.6	0.4	0.8	0.5	0.7	0.6			F=0.5	2, 96	0.6	—

*Scan 2*												
	*N*=19		*N*=29		*N*=42		*N*=45					
Age	24.3	3.8	25.7	4.2	25.5	4.3	24.8	3.4	F=0.7	3, 134	0.5	—
SANS totals	13.5	10.4	22.0	10.9	8.0	9.3			F=18.2	2, 89	<0.0001	1, 2>3
*Affective flattening*	5.7	5.9	7.8	5.3	2.2	3.5			*χ*^2^=23.6	2	<0.001	1, 2>3
*Alogia*	1.7	2.2	2.4	2.7	1.0	1.9			*χ*^2^=8.3	2	0.015	2>3
*Avolition/apathy*	5.9	3.7	4.9	4.0	1.7	2.4			F=14.2	2, 89	<0.001	1, 2>3
*Anhedonia/asocality*	6.6	4.2	6.9	4.4	3.1	3.4			F=10.0	2, 89	<0.001	1, 2>3
SAPS totals	5.9	6.2	13.5	10.4	3.5	8.6			*χ*^2^=24.7	2	<0.0001	2>1, 3
CDSS	1.0	1.5	1.8 [28]	3	1.4	2.7			*χ*^2^=1.4	2	0.5	—
HARS	3.8	6.0	4.9 [27]	4.3	2.0	2.8			*χ*^2^=8.12	2	0.017	2>3
CPZ equivalent (in mg)	2875.2	2059.7	4378.7	3291.3	2652.4	2160.4			*χ*^2^=10.5	2	0.005^b^	—
Adherence (%)	87.0	16.0	78.1	22	81.2	25.2			*χ*^2^=1.9	2	0.4	—
|Scan - Symptom Eval| (mo)	1.8	1.5	2	1.7	1.8	1.2			*χ*^2^=0.2	2	0.9	—

*Scan 3*												
	*N*=20		*N*=18		*N*=30		*N*=31					
Age	25.4	3.7	26.5	3.8	26.2	4.4	26.9	3.3	F=0.6	3, 98	0.6	—
SANS totals	19.8	11.1	17.2	16.5	6.7	8.5			F=8.6	2, 67	<0.0001	1, 2>3
*Affective flattening*	5.4	5.5	6.6	6.6	1.7	3.0			F=6.4	2, 67	0.003	1, 2>3
*Alogia*	2.9	3.0	2.0	3.2	1.0	1.8			*χ*^2^=6.0	2	0.05	1>3
*Avolition/apathy*	5.2	2.9	3.7	4.2	1.6	2.2			*χ*^2^=15.1	2	0.001	1, 2>3
*Anhedonia/asocality*	6.4	3.9	4.9	4.7	2.4	3.1			*χ*^2^=14.4	2	0.001	1, 2>3
SAPS totals	7.0	10.3	15.2	20.6	4.6	8.0			*χ*^2^=4.5	2	0.09	—
CDSS	2.5 [18]	3.3	2.3	2.6	1.5 [28]	2.1			*χ*^2^=1.2	2	0.6	—
HARS	3.0 [19]	4.3	5.9	7.6	2.8 [28]	3.4			*χ*^2^=1.98	2	0.371	—
CPZ equivalent (in mg)	4216.5	3906.2	6540.4	6243.9	5060.3	4948.9			*χ*^2^=1.8	2	0.4	—
Adherence (%)	78.3	26.2	75	30.5	77.9	28.6			*χ*^2^=0.07	2	0.97	—
|Scan - Symptom Eval| (mo)	1.0	1.9	0.5	0.7	0.4	0.7			*χ*^2^=0.7	2	0.7	—

Abbreviations: CDSS, Calgary Depression Scale for Schizophrenia; CPZ, chlorpromazine; DUI, Duration of Untreated Illness; DUP, Duration of Untreated Psychosis; ePNS, early persistent negative symptoms; FUP, follow-up; HARS, Q5 Hamilton Anxiety Rating Scale; IQ, intelligent quotient; NOS, Not Otherwise Specified; SAPS/SANS, scale for assessment of positive/negative symptoms; SCZ/SCZ-F, Schizophrenia/Schizophreniform; sPNS, persistent negative symptoms due to secondary factors.

a IQ means are presented as adjusted means, covaried by test version (WAIS-III versus WASI). There was no difference between different test versions on IQ (F_1,142_=1.15, *P*=0.29).

b *Post hoc* analyses indicated that sPNS patients were prescribed significantly more antipsychotic medication (in CPZ equivalent dosage) cumulatively compared with non-PNS patients at Scan 2 (*P*<0.05). Further tests revealed that when taking into consideration medication adherence (multiplying CPZ equivalent by percent adherence), only a trend-like difference existed between groups (*χ*^2^_(2)_=5.0, *P*=0.08). No difference between ePNS and the other two FEP subgroups at Scan 2 (*P*'s>0.05).

General demographics for whole sample are presented, followed by information corresponding to each scan. Scan 1 was conducted around baseline, Scan 2 represents 1-year follow-up and Scan 3 represents 2-year follow-up. All data represented as mean (s.d.), unless otherwise specified. Square brackets [] include adjusted sample size included in statistical analysis due to missing data points. Note: all antipsychotic totals are presented as cumulative chlorpromazine equivalents. SAPS/SANS totals are presented as mean scores of the sum of item-level scores from each scale; SANS totals exclude the ‘attention’ subscale. Item-level scores for each of the four SANS subscales per time point are italicized. *Post hoc* analyses were thresholded at *P*<0.05, and as follows: 1=ePNS; 2=sPNS; 3=Non-PNS; 4=Controls. For each time point, the absolute time in months between the date of scan and date of symptom evaluation was recorded, and displayed in the table as ‘|Scan—Symptom Eval|(mo)’.

**Table 2:** Generalized estimating equations statistics for negative and positive symptoms

	*ePNS*		*sPNS*		*Non-PNS*		*Omnibus*^a^		Post hoc^b^
	N	*Mean (s.d.)*	N	*Mean (s.d.)*	N	*Mean (s.d.)*	χ^*2*^_*(2)*_	P	
*2*									
SANS	20	24.85 (10.58)	30	21.67 (10.62)	44	15.52 (9.59)	14.29	<0.0001	ePNS, sPNS>non-PNS
SAPS		9.05 (11.69)		10.23 (10.63)		4.64 (6.72)	8.16	0.017	sPNS>non-PNS
									
*3*									
SANS	21	26.76 (9.34)	30	19.7 (9.40)	43	14.5 (10.96)	22.36	<0.0001	ePNS>sPNS>non-PNS
SAPS		7.14 (8.34)		9.7 (9.97)		3.44 (4.92)	12.59	0.002	sPNS>non-PNS
									
*6*									
SANS	18	26.39 (11.52)	30	22.23 (11.14)	41	11.41 (9.34)	34.57	<0.0001	ePNS, sPNS>non-PNS
SAPS		5.33 (5.70)		16.7 (11.52)		2.59 (3.64)	44.98	<0.0001	sPNS>ePNS, non-PNS
									
*9*									
SANS	18	22.28 (10.62)	28	20.18 (9.84)	45	11.13 (9.87)	23.1	<0.0001	ePNS, sPNS>non-PNS
SAPS		5.89 (6.33)		13.75 (14.18)		3.53 (6.68)	13.64	0.001	sPNS>ePNS, non-PNS
									
*12*									
SANS	20	23.65 (13.24)	27	24.82 (14.46)	46	8.17 (8.68)	46.85	<0.0001	ePNS, sPNS>non-PNS
SAPS		5.35 (5.26)		20.07 (15.39)		3.61 (7.16)	28.42	<0.0001	sPNS>ePNS, non-PNS
									
*18*									
SANS	20	18.95 (11.28)	29	22.48 (11.61)	43	6.84 (7.43)	53.41	<0.0001	ePNS, sPNS>non-PNS
SAPS		5.20 (5.34)		13.48 (13.31)		3.56 (9.01)	12.92	0.002	sPNS>ePNS, non-PNS
									
*24*									
SANS	20	17.15 (12.22)	23	16.74 (12.04)	38	8.40 (8.66)	14.07	0.001	ePNS, sPNS>non-PNS
SAPS		6.1 (9.20)		6.44 (5.97)	37	5.95 (12.37)	0.052	0.98	—

Abbreviations: ePNS, early persistent negative symptoms; GEE, generalized estimating equation; PEPP, prevention and early-intervention program for psychoses; SANS, scale for the assessment of negative symptoms; SAPS, scale for the assessment of positive symptoms; sPNS, persistent negative symptoms due to secondary factors.

a For global omnibus tests, there was a significant main effect of group (***χ***^**2**^_**(2)**_=65.31, *P*<0.001), time (***χ***^**2**^_(6)_=24.02, *P*<0.001), and group×time interaction (***χ***^2^_**(12)**_=32.20, *P*=0.001) on SANS scores. GEE analyses of SAPS scores also revealed a significant main effect of group (***χ***^**2**^_**(2)**_=40.03, *P*<0.0001), and group×time interaction (***χ***^**2**^_**(12)**_=34.69, *P*=0.001). No significant effect of time was found for SAPS scores. Omnibus results presented are ‘cross-sectional’ per time point, for ease of understanding.

b Bonferroni corrected, *P*<0.025.

Negative and positive symptoms represent sum of item-level scores as assessed by the SANS and SAPS, respectively. Note: SANS total excludes the ‘attention’ subscale. Left-hand column organizes statistics by clinical visit, relative to entry to PEPP-Montreal clinic (i.e., 2/3/6/9/12/18/24-month visits).
