# Supplementary material for: Age-related cortical thickness trajectories in first episode psychosis patients presenting with early persistent negative symptoms
Source: NPJ Schizophr. 2016 Aug 24;2:16029–. doi: 10.1038/npjschz.2016.29 (PMC5007985; doi:10.1038/npjschz.2016.29)
Supplement: Supplementary Information [file npjschz201629-s1.doc]

Additional MRI Quality Control (QC) Information

*QC.* All raw T1-weighted scans were visually inspected and quality controlled by three independent raters and scans were excluded if they exhibited excessive movement within the scanner, or the scan contained incidental findings. Scans that passed initial raw QC were then submitted to the CIVET pipeline. All CIVET outputs were quality controlled using the CBRAIN platform[[1]](#footnote-2) of which significant mask errors and/or minor pipeline errors were corrected through in-house scripts, if feasible. Specifically, inaccurate brain extractions (impacting 18 scans, corresponding to 10 FEP patients and 4 controls) were corrected by creating a new brain mask with *mincbet* within the minc-toolkit (<http://www.bic.mni.mcgill.ca/ServicesSoftware/ServicesSoftwareMincToolKit>), and applying this altered brainmask in subsequent stages of the CIVET pipeline. Inaccurate extraction of gray/white matter surfaces in close proximity to the posterior horn of the lateral ventricle due to a gradient error were corrected via in-house scripts (21 scans total, belonging to 13 FEP patients and 1 control). Surfaces were then re-run through specific stages of CIVET corresponding to the stage at which the error was found. Four scans did not pass through post-processing QC and corrections due to failure through the CIVET pipeline (1), and incidental findings (3 scans, belonging to one control).

**Supplementary Tables & Figures**

**A.** i. ePNS < Non-ePNS (all)* ii. ePNS < Controls iii. Non-ePNS (all) < Controls

(136df) (135df ) (139df)


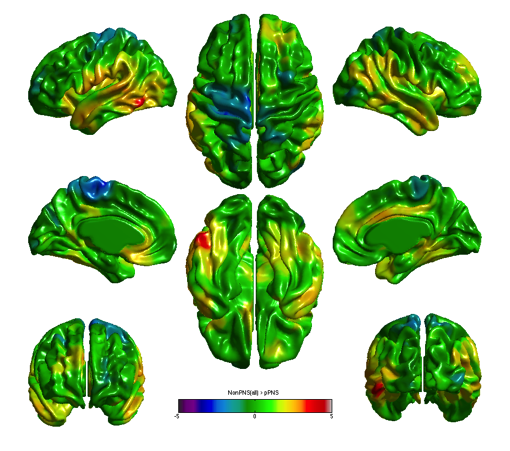

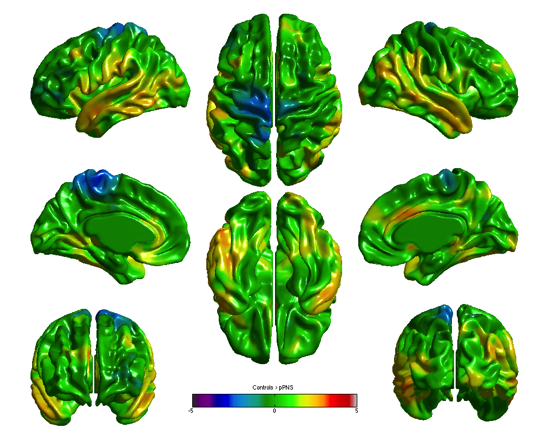

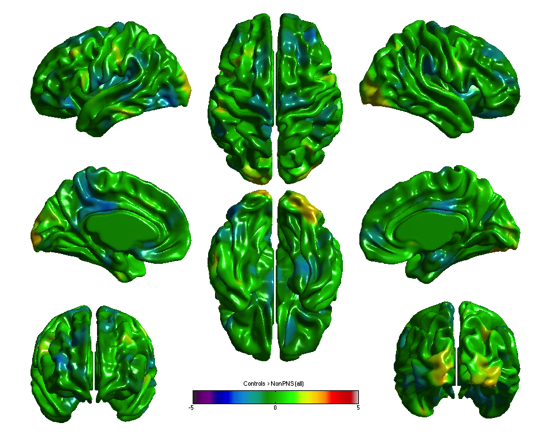


**B.** iv. sPNS > ePNS v. Non-PNS > sPNS

(136df) (138df)

**
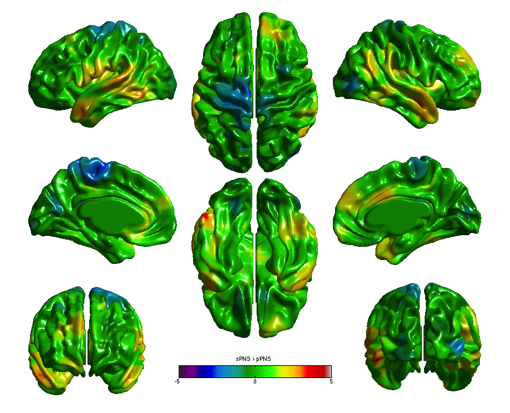

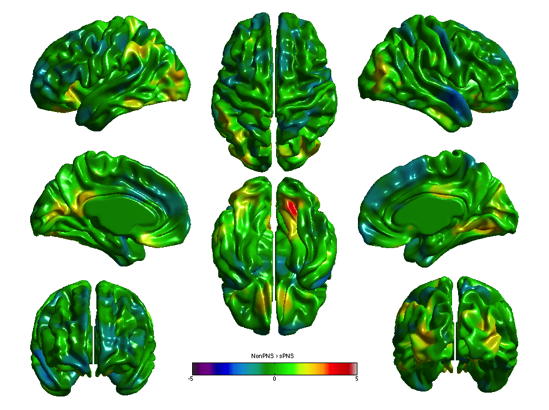
**

**Supplementary Figure 1**. t-statistic maps for main effect of group. Top row (**A**), depicts contrasts with entire non-ePNS sample (including sPNS), i-iii. Bottom row (**B**), further explores cortical thickness differences between sPNS and iv. ePNS, and v. remaining Non-PNS patients. The asterisk(*) indicates the only significant group contrast, as discussed in the main manuscript.

Abbreviations: ePNS, early persistent negative symptoms. sPNS, persistent negative symptoms due to secondary factors.

i. ePNS* ii. sPNS* iii. Non-PNS iv. Controls

(50df) (67df) (97df) (112df)


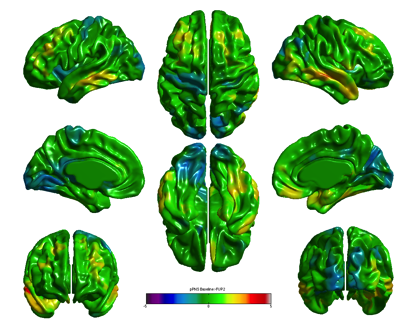

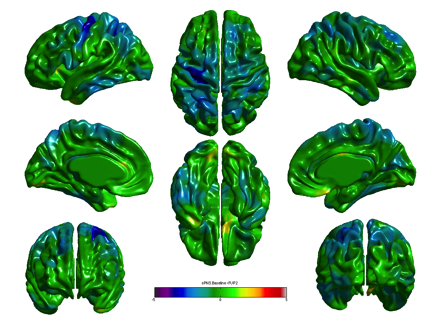

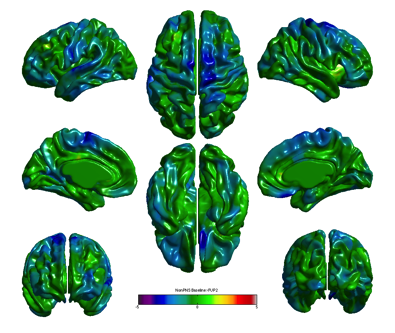

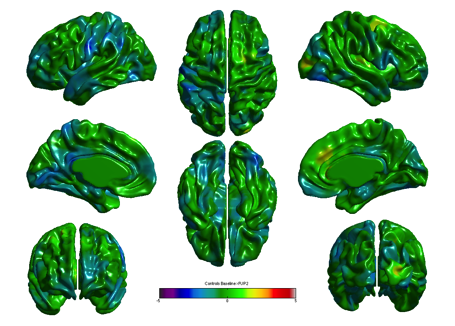


**Supplementary Figure 2**. t-statistic maps for main effect of time. All contrasts shown as Baseline>FUP2. Warm colours (positive t-values) represent cortical thinning from Baseline to FUP2, whereas cool colours (negative t-values) represent increased cortical thickness from Baseline to FUP2. Asterisks(*) represent significant time effects on cortical thickness, as presented in the main manuscript.

**Abbreviations**: FUP2, follow-up year two; ePNS, early persistent negative symptoms. sPNS, persistent negative symptoms due to secondary factors.

**A.** i. ePNS > Non-ePNS (all)* ii. ePNS > Controls iii. Non-ePNS (all) > Controls

(247df) (249df) (232df)


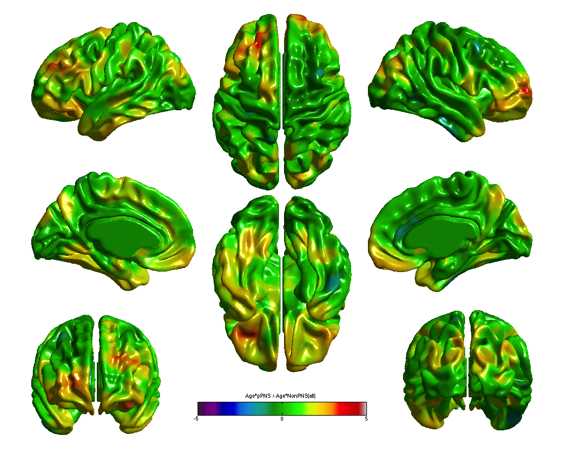

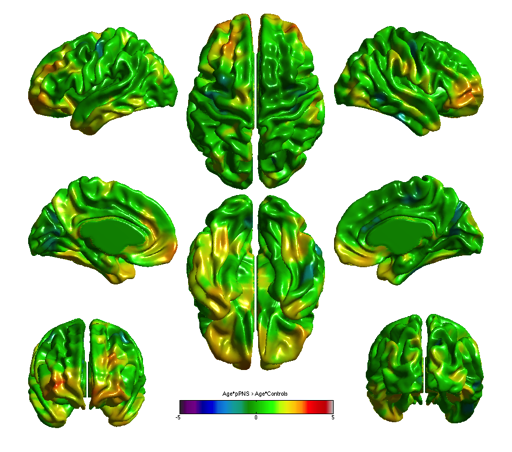

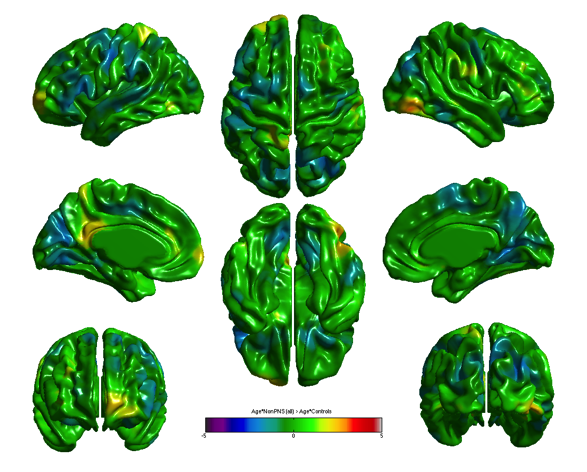


**B.** iv. ePNS > sPNS* v. sPNS > Non-PNS**

(238df) (213df)


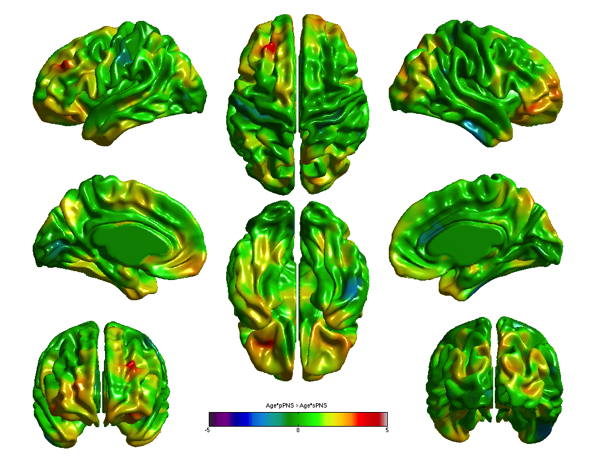

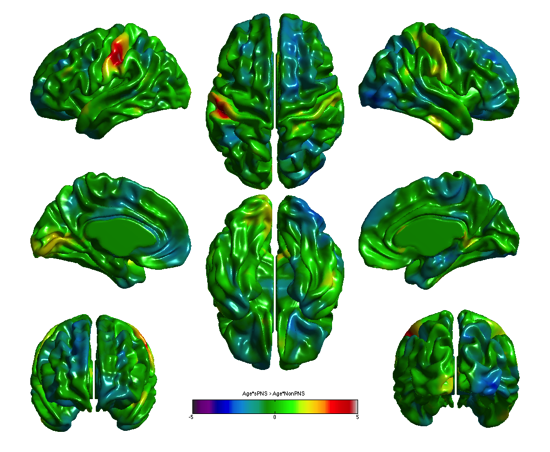


**Supplementary Figure 3**. t-statistic maps for age by group interaction, with linear effects of age. Top row (**A**), depicts contrasts of age by group interaction with entire non-ePNS sample (including sPNS), i-iii. Bottom row (**B**), further explores cortical thickness differences with linear age effects between sPNS and iv. ePNS, and v. remaining non-PNS patients. A single asterisk(*) represents significant age by group interactions, as presented in the manuscript. Double asterisks (**) represents a significant age by group interaction effect between sPNS and remaining non-PNS patients, where a region of the left post central gyrus had a significantly different and positive age effect in sPNS compared to non-PNS.

**Abbreviations**: ePNS, early persistent negative symptoms. sPNS, persistent negative symptoms due to secondary factors.

**A.** i. ePNS > Non-ePNS (all)* ii. ePNS > Controls iii. Non-ePNS (all) > Controls

(353df) (354df) (351df)


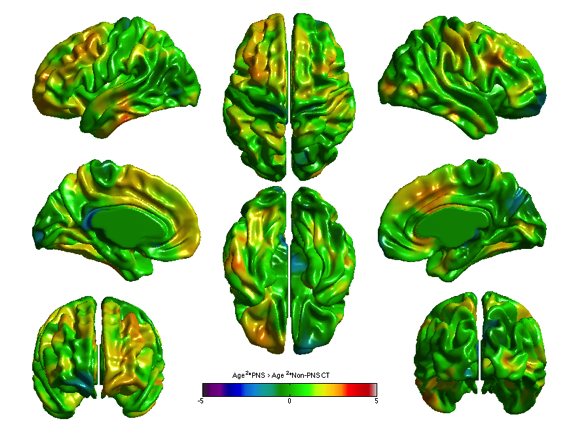

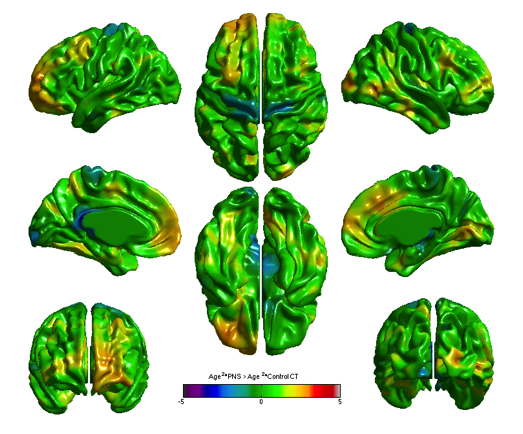

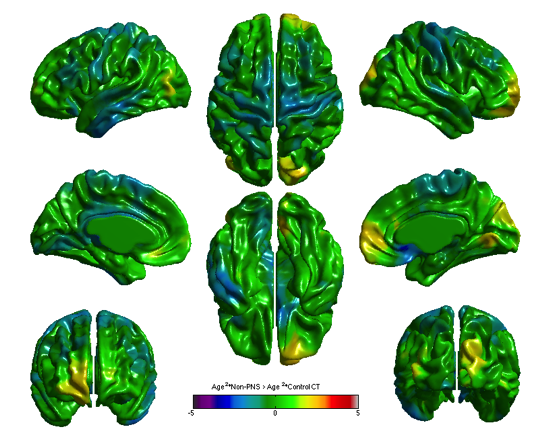


**B.** iv. ePNS > sPNS* v. sPNS > Non-ePNS

(348df) (327df)


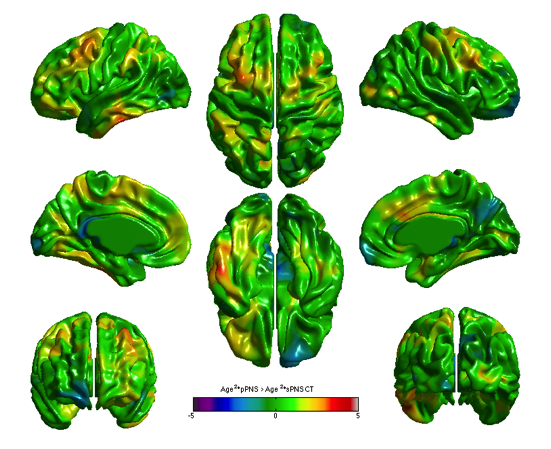

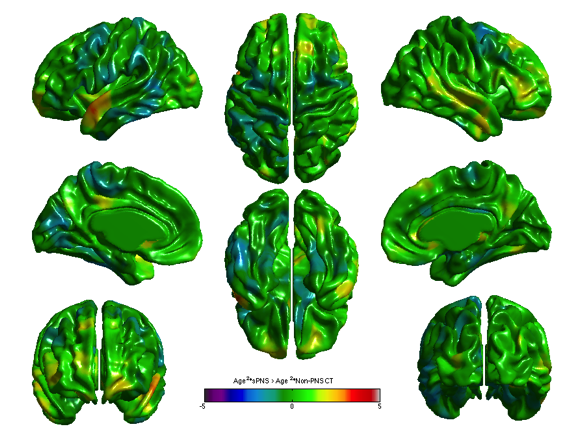


**Supplementary Figure 4**. t-statistic maps for age^2^ by group interaction, with quadratic effects of age. Top row (**A**), depicts contrasts of age^2^ by group interaction with entire non-ePNS sample (including sPNS), i-iii. Bottom row (**B**), further explores cortical thickness differences with quadratic age effects between sPNS and iv. ePNS, and v. remaining non-PNS patients. Asterisks (*) represent significant age^2^ by group interactions, as presented in the manuscript.

**Abbreviations:** ePNS, early persistent negative symptoms. sPNS, persistent negative symptoms due to secondary factors.

| **Effect** | **Cortical Region** | **Manuscript Figure** | **ePNS** | **sPNS** | **Non-PNS** | **Controls** |
| --- | --- | --- | --- | --- | --- | --- |
| Scan Time (age controlled) | R Middle Temporal | Figure 2B | -0.71 | -0.20 | 0.00 | -0.38 |
|  | L Pre/Post-Central | Figure 2C | 0.47 | 1.14 | 0.59 | -0.36 |
|  |  |  |  |  |  |  |
| Age*Group (Linear) | L DLPFC | Figure 3A | 0.50 | -0.38 | -0.24 | -0.29 |
|  | L OFC | Figure 3B | 0.30 | -0.39 | -0.30 | -0.30 |
|  | R Anterior Frontal | Figure 3C | 0.30 | -0.40 | -0.30 | -0.30 |

**Supplementary Table 1.** Cortical thickness percent change per year, for ROIs showing linear change over time, as detailed in main manuscript. Top half of table compares percent change per year over the two-year follow-up period for regions showing significant time effects (Figure 3 of main manuscript), and controlling for age. Bottom half of table quantifies percent change per year for regions showing linear effects of age (Figure 3 of main manuscript). Negative values represent percent cortical thinning per year, whereas positive values represent increased cortical thickness.

**Abbreviations**: L, Left; R, Right; DLPFC, dorsolateral prefrontal cortex; OFC, orbitofrontal cortex; ePNS, early persistent negative symptoms. sPNS, persistent negative symptoms due to secondary factors.

| **Cortical Region** | **Manuscript Figure** | **Significant Term** | **Linear Age Term** | **Quadratic Age Term** |
| --- | --- | --- | --- | --- |
| L DLPFC | Figure 3A | Age*Group (Linear) | -256.53 | -253.16 |
| L OFC | Figure 3B | Age*Group (Linear) | -279.7 | -275.9 |
| R Anterior Frontal | Figure 3C | Age*Group (Linear) | -196.88 | -193.6 |
| L DLPFC/pre-SMA | Figure 4A | Age^2^*Group (Quadratic) | -589.52 | **-592.47** |
| R Middle Cingulate | Figure 4B | Age^2^*Group (Quadratic) | -545.9 | **-549.76** |
| L Inferior Temporal | Figure 4C | Age^2^*Group (Quadratic) | -411.8 | **-417.59** |

**Supplementary Table 2.** Akaike Information Criterion (AIC) values comparing linear and quadratic age terms for significant cortical regions which emerged from vertex-wise analyses of age*group interactions. Significant likelihood ratio tests are bolded, indicating more complex quadratic term was a better fit to the data. Otherwise, a more simplistic (linear) model was used. Note, the Linear Age Term includes "Age" and "Symptoms*Age"**.** The Quadratic Age Term includes "Age", "Age^2^", "Symptoms*Age", and "Symptoms*Age^2^".

**Abbreviations:** L, Left; R, Right; DLPFC, dorsolateral prefrontal cortex; OFC, orbitofrontal cortex; pre-SMA, pre- Supplementary Motor Area.

|  |  |  | Original Statistics | | | Controlling for Antipsychotic Medication | | |  |
| --- | --- | --- | --- | --- | --- | --- | --- | --- | --- |
| **Cortical Region** | **Manuscript Figure** | **Significant Term** | **F-statistic** | **df** | **p-value** | **F-statistic** | **df** | **p-value** | **post-hoc** |
| L Inferior Temporal | Figure 2A | Group | 6.89 | 1, 248 | 0.0012 | 6.91 | 1, 247 | 0.0012 | ePNS<sPNS, non-PNS |
| R Middle Temporal | Figure 2B | Scan Time, ePNS | 7.40 | 2, 53 | 0.0015 | 6.95 | 2, 52 | 0.0021 | Baseline>FUP2 |
| L Pre/Post-Central | Figure 2C | Scan Time, Non-PNS | 8.74 | 2, 188 | 0.00023 | 8.88 | 2, 187 | 0.00021 | Baseline<FUP1>  FUP2 |
| L DLPFC | Figure 3A | Age*Group (Linear) | 5.92 | 2, 246 | 0.0031 | 5.91 | 2, 245 | 0.0031 | ePNS>sPNS |
| L OFC | Figure 3B | Age*Group (Linear) | 5.33 | 2, 246 | 0.0054 | 5.70 | 2, 245 | 0.0038 | ePNS>sPNS |
| R Anterior Frontal | Figure 3C | Age*Group (Linear) | 4.78 | 2, 246 | 0.0092 | 4.96 | 2, 245 | 0.0077 | ePNS>sPNS |
| L DLPFC/ pre-SMA | Figure 4A | Age^2^*Group (Quadratic) | 4.51 | 2, 243 | 0.033 | 4.65 | 2, 242 | 0.010 | ePNS>sPNS |
| R Middle Cingulate | Figure 4B | Age^2^*Group (Quadratic) | 4.02 | 2, 243 | 0.019 | 4.03 | 2, 242 | 0.019 | ePNS<sPNS, non-PNS |
| L Inferior Temporal | Figure 4C | Age^2^*Group (Quadratic) | 6.57 | 2, 243 | 0.0017 | 6.68 | 2, 242 | 0.0015 | ePNS>sPNS |

**Supplementary Table 3.** Mixed effects models applied to mean cortical thickness of each significant cortical region uncovered by initial vertex-wise analyses, comparing initial model presented in manuscript, and a similar model including antipsychotic medication as a covariate. All analyses controlled for gender, handedness and proxy BV as specified in main manuscript (and age when this was not a main effect of interest). Antipsychotic medication represented as cumulative chlorpromazine equivalent, multiplied by medication adherence. Given that Controls do not take such medication, controls were not included in the models presented above.

^1^Antipsychotic medication had a significant effect on cortical thickness within left OFC for linear age effect, but symptoms*age interaction remained significant even after controlling for medication.

**Abbreviations.** L, Left; R, Right; DLPFC, dorsolateral prefrontal cortex; OFC, orbitofrontal cortex; pre-SMA, pre- Supplementary Motor Area; ePNS, early persistent negative symptoms. sPNS, persistent negative symptoms due to secondary factors; FUP1/2=Follow-Up Year 1/2

1. Sherif T, Rioux P, Rousseau M-E, Kassis N, Beck N, Adalat R, Das S, Glatard T, Evans AC (2014) CBRAIN: a web-based, distributed computing platform for collaborative neuroimaging research. NeuroImage 8:54. [↑](#footnote-ref-2)
